# Novel Orange Color Pigments Based on La_3_LiMnO_7_

**DOI:** 10.3390/molecules26206243

**Published:** 2021-10-15

**Authors:** Ryohei Oka, Jun-ichi Koyama, Takuro Morimoto, Toshiyuki Masui

**Affiliations:** 1 Field of Advanced Ceramics, Department of Life Science and Applied Chemistry, Graduate School of Engineering, Nagoya Institute of Technology, Gokiso, Showa, Nagoya 466-8555, Aichi, Japan; oka.ryohei@nitech.ac.jp; 2 Department of Chemistry and Biotechnology, Faculty of Engineering, and Center for Research on Green Sus-tainable Chemistry, Tottori University, 4-101, Koyama-cho Minami, Tottori 680-8552, Japan; b15t3045h@gmail.com; 3 Department of Engineering, Graduate School of Sustainability Science, Tottori University, 4-101, Koyama-cho Minami, Tottori 680-8552, Japan; M21J5037H@edu.tottori-u.ac.jp

**Keywords:** inorganic pigments, orange color, environment-friendly, Mn^4+^ ion, d–d transition

## Abstract

La_3_LiMn_1−*x*_Ti*_x_*O_7_ (0 ≤ *x* ≤ 0.05) samples were synthesized by a solid-state reaction method, and a single-phase form was observed for the samples in the range of *x* ≤ 0.03. Crystal structure, optical properties, and color of the La_3_LiMn_1−*x*_Ti*_x_*O_7_ (0 ≤ *x* ≤ 0.03) samples were characterized. Strong optical absorption was observed at a wavelength between 400 and 550 nm, and a shoulder absorption peak also appeared around 690 nm in all samples; orange colors were also exhibited. Among the samples synthesized, the most brilliant orange color was obtained at La_3_LiMn_0.97_Ti_0.03_O_7_. The redness (a*) and yellowness (b*) values of this pigment were higher than those of the commercially available orange pigments. Therefore, the orange color of this pigment is brighter than those of the commercial products. Since the La_3_LiMn_0.97_Ti_0.03_O pigment is composed of non-toxic elements, it could be a new environmentally friendly inorganic orange pigment.

## 1. Introduction

Inorganic pigments have been widely applied to paints, glasses, ceramics, etc., because of their high hiding power and thermal stability compared to organic pigments [1]. Several orange pigments such as cadmium orange (CdS·CdSe), molybdate orange (PbCrO_4_·PbMoO_4_·PbSO_4_), and bayferrox orange (Fe_2_O_3_·FeOOH) are conventionally used. However, the use of the cadmium and molybdate orange pigments has been forbidden or restricted because they contain toxic elements which have harmful effects on the human body and the environment. Although the bayferrox orange pigment is environmentally friendly, the vividness of this pigment is not sufficient. Therefore, development of environmentally friendly inorganic orange pigments is required, and several studies have been reported [2,3,4,5,6,7,8,9,10,11,12,13,14,15,16,17]. Some compounds, such as La_2_Ce_2−*x*_W_0.5*x*_Fe_0.5*x*_O_7+*δ*_ [14], Sr_4_Mn_2_Cu_0.5_Zn_0.5_O_9_ [15], and SrBaCe_0.6_Tb_0.4_O_4_ [3], for example, have been proposed and exhibit an orange color. Unfortunately, the colors of La_2_Ce_2−*x*_W_0.5*x*_Fe_0.5*x*_O_7+*δ*_ and Sr_4_Mn_2_Cu_0.5_Zn_0.5_O_9_ are pale or dark, and SrBaCe_0.6_Tb_0.4_O_4_ contains Tb, for which raw materials are expensive. Hence, environment-friendly and low-cost orange pigments are desirable.

Because of this situation, we focused on Mn^4+^ as an orange coloring source. Mn^4+^ has been investigated as an activator for the red-light emitting phosphors [18,19,20,21,22,23,24,25]. These Mn^4+^-activated phosphors absorb/emit visible light, due to the d-d transition. In general, the absorption intensity and wavelength of the optical absorption band corresponding to the d-d transition are strongly influenced by the content of Mn^4+^ and the coordination environment around the Mn^4+^ ions, respectively. In the case of phosphors, the concentration of Mn^4+^ is limited by ≤1% mol, but the absorption becomes stronger as the Mn^4+^ concentration increases. Recently, the materials based on Li_2_MnO_3_ containing a large amount of Mn^4+^ have been reported as environment-friendly red pigments [26,27,28]. The pure Li_2_MnO_3_ pigment shows orange color, while the color becomes reddish by doping with other cations. In other words, Mn^4+^ ion is a promising coloring source for not only red but also orange.

In this study, we selected La_3_LiMnO_7_ as a host material for environmentally friendly orange pigment. This compound could have quite low toxicity as compared with the conventional pigments containing toxic elements, because toxicity of the constituent elements is quite lower than that of toxic ones, such as Cd and Pb. In addition, raw materials of this are cost-effective. The components of this material are similar to those of Li_2_MnO_3_. Therefore, it is expected that the host La_3_LiMnO_7_ material exhibit orange color due to the d–d transition of Mn^4+^. As mentioned above, the optical absorption of the d–d transition is influenced by the geometric structure around Mn^4+^ and the concentration of Mn^4+^. If other cations such as Ti^4+^ (ionic radius: 0.0605 nm [29]), the ionic radius of which is close to that of Mn^4+^ (ionic radius: 0.053 nm [29]), are doped into the Mn^4+^ site, it is possible to tune the color by controlling the content and geometric structure for Mn^4+^. For these reasons, the La_3_LiMn_1−*x*_Ti*_x_*O_7_ (0 ≤ *x* ≤ 0.05) samples were synthesized by a solid-state reaction method, and their optical and color properties were characterized as environmentally friendly inorganic orange pigments.

## 2. Results and Discussion

### 2.1. X-ray Powder Diffraction (XRD) and Scanning Electron Microscopy (SEM) Image

**Figure 1:**
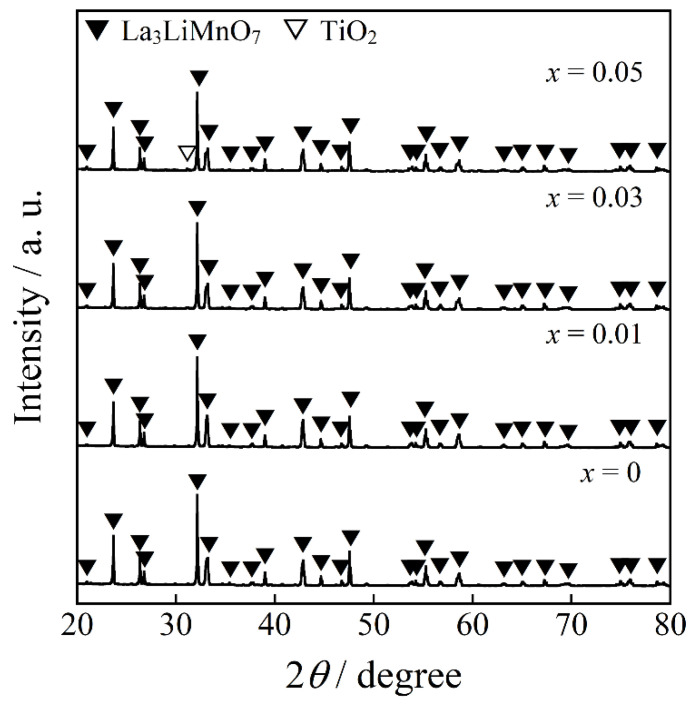
XRD patterns of the La_3_LiMn_1−*x*_Ti*_x_*O_7_ (0 ≤ *x* ≤ 0.05) samples.

Figure 1 shows the XRD patterns of the La_3_LiMn_1−*x*_Ti*_x_*O_7_ (0 ≤ *x* ≤ 0.05) samples. The positions, full width at half maximum (FWHM), and relative integrated intensities (RII) of diffraction peaks for La_3_LiMnO_7_ phase are also listed in Table 1. A single-phase form was observed for the samples in the range of *x* ≤ 0.03, and all diffraction peaks were assigned to the La_3_LiMnO_7_ phase. On the other hand, an impurity phase (TiO_2_) was detected in the sample with *x* = 0.05.

**Figure 2:**
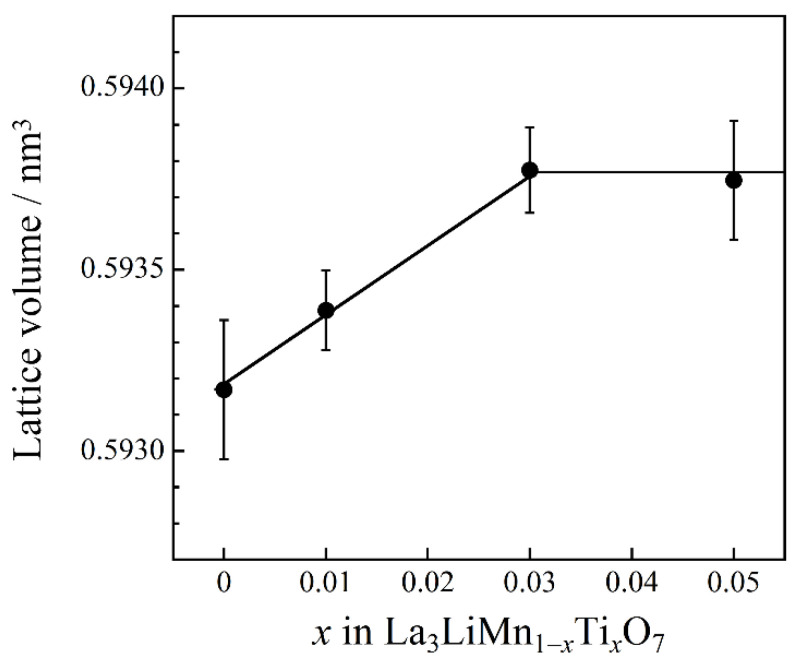
Compositional dependence of the lattice volume for La_3_LiMn_1−*x*_Ti*_x_*O_7_ (0 ≤ *x* ≤ 0.05).

La_3_LiMnO_7_ belongs to the layered perovskite type structure, and it crystallizes into a tetragonal structure with space group of *P*4_2_/*mnm* (No. 136) [30]. The Li^+^ and Mn^4+^ ions occupy one 8j site according to the Wyckoff notation. These cations form the [Li/MnO_6_] octahedra surrounded by six O^2−^ ions. Figure 2 shows the composition dependence of the lattice volume for the La_3_LiMn_1−*x*_Ti*_x_*O_7_ (0 ≤ *x* ≤ 0.05) samples. Cell volume increased as the Ti^4+^ content increased in the range of *x* ≤ 0.03. This result indicates that some Mn^4+^ (ionic radius: 0.053 nm [29]) ions in the host lattice were substituted with larger Ti^4+^ (ionic radius: 0.0605 nm [29]) ones. The lattice volumes of the samples for *x* = 0.03 and 0.05 were even equal. Therefore, the solubility limit of Ti^4+^ into the La_3_LiMnO_7_ lattice was *x* = 0.03.

**Figure 3:**
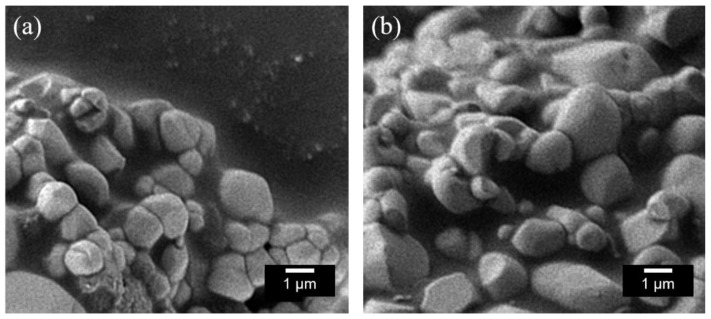
SEM images of (**a**) La_3_LiMnO_7_ and (**b**) La_3_LiMn_0.97_Ti_0.03_O_7_.

The SEM images of the La_3_LiMn_1−*x*_Ti*_x_*O_7_ (*x* = 0 and 0.03) samples are shown in Figure 3. The particle size was about 1 µm in both samples, and particle aggregation was observed. Colors of materials are affected by various factors such as crystal structure, chemical composition, and particle size. In both present samples, there was no significant change in particle size and morphology as seen in Figure 3. These results indicate that the change in color properties was attributed to the changes in crystal structure and optical absorption caused by doping Ti^4+^.

### 2.2. X-ray Fluorescence Analysis (XRF)

The element ratios of La, Mn, and Ti for the La_3_LiMn_1−*x*_Ti*_x_*O_7_ (0 ≤ *x* ≤ 0.03) samples, which were obtained in a single-phase form, were analyzed by XRF, and the results are summarized in Table 2. They were in approximate agreement with the stoichiometric ratios of the starting mixtures.

### 2.3. Optical Reflectance Spectra

**Figure 4:**
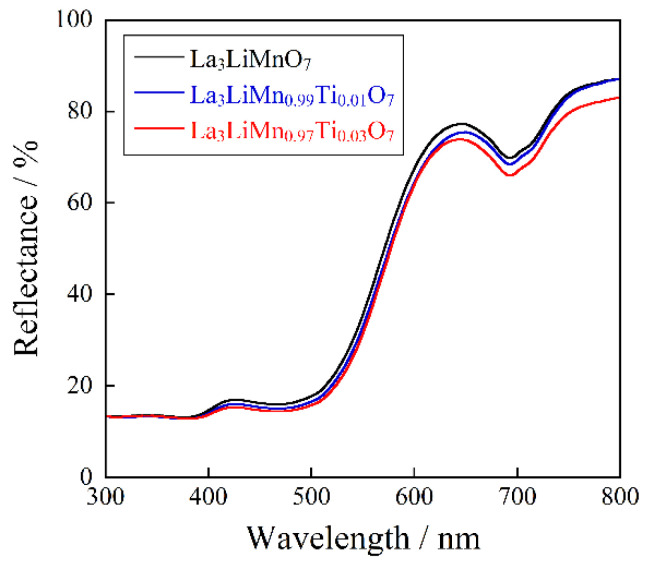
UV–Vis reflectance spectra for the La_3_LiMn_1−*x*_Ti*_x_*O_7_ (0 ≤ *x* ≤ 0.03) samples.

The UV–Vis reflectance spectra of the La_3_LiMn_1−*x*_Ti*_x_*O_7_ (0 ≤ *x* ≤ 0.03) samples, which were obtained in a single-phase form, are depicted in Figure 4. The optical absorption below 400 nm in the UV light region corresponded to the O_2p_-Mn_3d_ charge transfer transition, while those around 500 and 690 nm in the visible region were attributed to the d-d transition of Mn^4+^. The former was spin-allowed ^4^A_2g_–^4^T_2g_ transition and the latter was spin-forbidden ^4^A_2g_2212^2^E_g_ and ^4^A_2g_–^2^T_1g_ transitions, respectively [22,26,27,28].

The absorption intensity of the d-d transition bands was increased by doping with Ti^4+^. This behavior was due to the increased distortion of the [MnO_6_] octahedra, which was caused by the Ti^4+^ substitution, because Ti^4+^ (0.0605 nm [29]) was larger than Mn^4+^ (0.053 nm [29]). Although the d-d transitions were essentially forbidden, they were partially allowed due to the loss of symmetry. As a result, the absorption intensity of the d-d transitions was increased by the Ti^4+^ doping.

**Figure 5:**
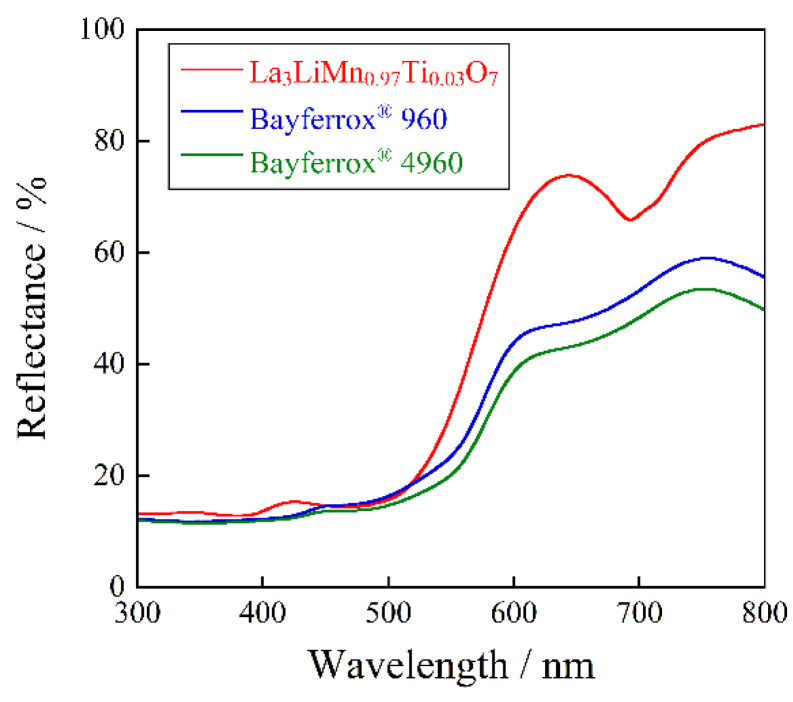
UV–Vis reflectance spectra for La_3_LiMn_0.97_Ti_0.03_O_7_, Bayferrox^®^ 960 and Bayferrox^®^ 4960.

The UV–Vis reflectance spectrum of La_3_LiMn_0.97_Ti_0.03_O_7_ was compared to those of the commercial orange pigments such as Bayferrox^®^ 960 and Bayferrox^®^ 4960 (Fe_2_O_3_-FeOOH, Ozeki), as shown in Figure 5. The reflectance values in the green-blue light region (480–490 nm), corresponding to the complementary color of orange, of the current La_3_LiMn_0.97_Ti_0.03_O_7_ and commercial pigments were almost the same. On the other hand, the La_3_LiMn_0.97_Ti_0.03_O_7_ pigment showed higher reflectance in the yellow-red light region (580–750 nm) than those of the conventional ones. Accordingly, the La_3_LiMn_0.97_Ti_0.03_O_7_ pigment exhibited more vibrant orange color than the commercially available orange pigments.

### 2.4. Color Properties

**Figure 6:**
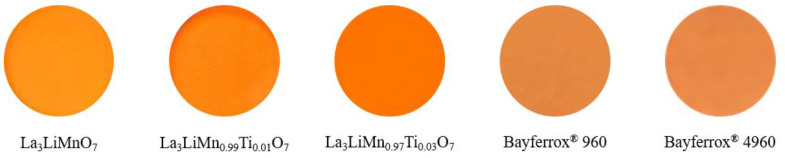
Photographs of the La_3_LiMn_1−*x*_Ti*_x_*O_7_ (0 ≤ *x* ≤ 0.03) and commercially available orange Bayferrox^®^ 960 and Bayferrox^®^ 4960 pigments.

The L*a*b*Ch° color coordinate data of the La_3_LiMn_1−*x*_Ti*_x_*O_7_ (0 ≤ *x* ≤ 0.03) and commercially available orange Bayferrox^®^ 960 and Bayferrox^®^ 4960 pigments are summarized in Table 3 (See Section 3.2 described in later for the detail of these parameters). The photographs of these orange pigments are also shown in Figure 6. The hue angle (*h*°) values of the samples synthesized in this study have fallen within the orange color region (35°–70°). The redness (a*), yellowness (b*), and chroma (*C*) values were slightly increased by doping with Ti^4+^ into the Mn^4+^ site. As already discussed above on the results in Figure 4, the optical absorption from 480 to 490 nm (green-blue) became stronger when the Ti^4+^ ions were introduced into the Mn^4+^ site in the host lattice. Therefore, the sample color became more vivid orange.

As recognized in Table 3, the *C* value of the present La_3_LiMn_0.97_Ti_0.03_O_7_ pigment was much higher than those of the commercial orange pigments. In addition, the *h*° value of these pigments were almost equivalent. These results elucidate that the La_3_LiMn_0.97_Ti_0.03_O_7_ pigment exhibited a bright orange color with high color purity, compared to the commercial orange pigments.

### 2.5. Chemical Stability Test

**Figure 7:**
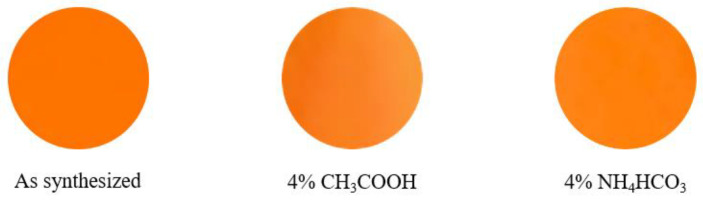
Photographs of the La_3_LiMn_0.97_Ti_0.03_O_7_ pigment before and after the chemical stability test.

The chemical stability of the La_3_LiMn_0.97_Ti_0.03_O_7_ pigment was tested in the acid/base solutions. The pigment was dispersed into the 4% acetic acid and 4% ammonium bicarbonate solutions. These sample solutions for the acid and base conditions were left at room temperature for 1 and 24 h. After that, the samples were washed with deionized water and ethanol, and then dried at room temperature. The color of the pigment after the chemical stability test was evaluated by using the colorimeter. The L*a*b*Ch° color coordinate data of the La_3_LiMn_0.97_Ti_0.03_O_7_ pigment before and after the soaking test are tabulated in Table 4, and the photographs are also displayed in Figure 7. After the soaking test, the b* value decreased slightly, but the *h*° value was almost constant and no significant color degradation was observed.

## 3. Materials and Methods

### 3.1. Synthesis

The La_3_LiMn_1−*x*_Ti*_x_*O_7_ (0 ≤ *x* ≤ 0.05) samples were synthesized by a conventional solid state reaction method. La_2_O_3_ (99.99%), Li_2_CO_3_ (99.0%), Mn_2_O_3_ (99.0%), and TiO_2_ (99.0%) powders were used as starting reagents. Stoichiometric amounts of metal oxides and three times the stoichiometric amount of Li_2_CO_3_ were mixed in an agate mortar. The homogeneous mixtures were calcined in an alumina crucible at 900 °C for 6 h in air. Before characterization, the samples were ground in an agate mortar.

### 3.2. Characterization

The element ratios of La, Mn, and Ti for the samples were confirmed by using X-ray fluorescence spectroscopy (XRF; Rigaku, ZSX Primus). The crystal structure of the samples was identified by X-ray powder diffraction (XRD; Rigaku, Ultima IV) with Cu-Kα radiation, operating with voltage and current settings of 40 kV and 40 mA. The lattice parameters and volumes were calculated from the XRD peak angles refined, using α-Al_2_O_3_ as a standard and using the CellCalc Ver. 2.20 software. The morphologies and particle sizes were observed by using field-emission-type scanning electron microscopy (FE-SEM; JEOL, JSM-6701F).

The optical reflectance spectra were measured with a UV-Vis-NIR spectrometer (JASCO, V-770), using a standard white plate as a reference. The color properties were evaluated in terms of the Commission Internationale de l’Éclairage (CIE) L*a*b*Ch° system, using a colorimeter (Konica-Minolta, CR-300). The L* parameter represents the brightness or darkness in a neutral grayscale. The a* and b* values indicate the red–green and yellow-blue axes, respectively. The chroma parameter (*C*) expresses the color saturation and is calculated with the formula, *C* = [(a*)^2^ + (b*)^2^]^1/2^. The hue angle (*h*°) ranges from 0 to 360° and is estimated according to the following formula: *h*° = tan^−1^(b*/a*).

## 4. Conclusions

La_3_LiMn_1−*x*_Ti*_x_*O_7_ (0 ≤ *x* ≤ 0.05) samples were synthesized using a solid-state reaction technique as environmentally friendly inorganic orange pigments. The La_3_LiMn_1−*x*_Ti*_x_*O_7_ (*x* = 0, 0.01, and 0.03) samples were obtained in a single-phase form, but an impurity phase was observed for *x* = 0.05. In the visible light region, the optical absorption band at a wavelength below 550 nm and the shoulder absorption peak around 690 nm were attributed to the spin-allowed and spin-forbidden d–d transitions of Mn^4+^, respectively. These absorption intensities were increased by the Ti^4+^ doping, because the [MnO_6_] octahedra were more distorted. Accordingly, the sample color became more vivid orange. Among the samples synthesized in this study, the La_3_LiMn_0.97_Ti_0.03_O_7_ pigment exhibited the most brilliant orange color. In addition, the orange color of the present pigment was brighter than those of the commercially available orange pigments, because the a* and b* values of this pigment were higher than those of the commercial ones. Since the orange color of the La_3_LiMn_0.97_Ti_0.03_O_7_ pigment has chemical stability, it has a potential to be a novel environmentally friendly inorganic orange pigment.

## Figures and Tables

**Table 1:** Peak positions, full width at half maximum (FWHM), and relative integrated intensities (RII) of diffraction peaks for La_3_LiMnO_7_ phase.

*x* = 0			*x* = 0.01			*x* = 0.03			*x* = 0.05		
2*θ*/deg.	FWHM	RII/%	2*θ*/deg.	FWHM	RII/%	2*θ*/deg.	FWHM	RII/%	2*θ*/deg.	FWHM	RII/%
21.00	0.12	4	20.99	0.48	6	20.98	0.40	11	20.99	0.08	4
23.67	0.06	51	23.67	0.07	50	23.67	0.07	51	23.67	0.07	50
26.36	0.07	28	26.36	0.07	25	26.37	0.07	27	26.36	0.07	25
26.78	0.07	15	26.77	0.07	15	26.77	0.07	15	26.77	0.07	14
32.16	0.07	100	32.16	0.07	100	32.16	0.07	100	32.16	0.07	100
33.07	0.19	47	33.11	0.22	69	33.04	0.17	39	33.22	0.23	54
35.41	0.05	2	35.38	0.11	3	35.41	0.08	2	35.39	0.06	2
37.70	0.24	7	37.66	0.20	8	37.69	0.27	7	37.61	0.19	6
38.99	0.07	15	38.99	0.08	15	38.99	0.08	15	38.99	0.08	16
42.76	0.14	30	42.82	0.18	55	42.73	0.13	27	42.73	0.13	30
44.66	0.07	11	44.66	0.08	10	44.67	0.08	10	44.66	0.08	10
46.77	0.08	5	46.77	0.09	5	46.77	0.09	5	46.77	0.08	5
47.54	0.08	42	47.53	0.09	42	47.52	0.08	41	47.52	0.08	42
53.72	0.29	10	53.76	0.30	13	53.67	0.27	11	53.67	0.31	10
54.25	0.08	5	54.24	0.08	5	54.25	0.08	6	54.25	0.08	5
55.17	0.08	17	55.16	0.09	16	55.15	0.09	19	55.15	0.10	19
55.29	0.09	21	55.29	0.09	21	55.29	0.09	20	55.29	0.08	18
56.76	0.21	10	56.71	0.17	9	56.77	0.21	9	56.65	0.13	5
58.49	0.22	21	58.60	0.27	36	58.40	0.26	27	58.41	0.27	26
63.21	0.32	7	63.14	0.29	8	63.15	0.39	9	63.22	0.25	5
65.07	0.20	9	65.05	0.17	9	65.01	0.21	10	65.03	0.22	9
67.27	0.11	9	67.26	0.10	10	67.25	0.11	10	67.26	0.11	10
69.44	0.72	11	69.40	0.29	6	69.27	0.51	8	69.28	0.63	9
74.99	0.13	9	74.97	0.13	9	74.94	0.13	10	74.94	0.12	9
75.96	0.32	24	75.87	0.31	23	75.74	0.37	22	75.68	0.21	10
78.64	0.12	7	78.62	0.12	7	78.62	0.14	7	78.60	0.13	7
79.07	0.37	7	79.10	0.39	13	79.16	0.48	10	78.97	0.32	6

**Table 2:** Elemental ratios of La, Mn, and Ti for the La_3_LiMn_1−*x*_Ti*_x_*O_7_ (0 ≤ *x* ≤ 0.03) samples.

Samples	Stoichiometry (La:Mn:Ti)	Analyzed Ratio (La:Mn:Ti)
La_3_LiMnO_7_	3:1	3.03:0.97
La_3_LiMn_0.99_Ti_0.01_O_7_	3:0.99:0.01	3.01:0.96:0.03
La_3_LiMn_0.97_Ti_0.03_O_7_	3:0.97:0.03	3.01:0.94:0.05

**Table 3:** L*a*b*Ch° color coordinate data of the La_3_LiMn_1−*x*_Ti*_x_*O_7_ (0 ≤ *x* ≤ 0.03) and commercially available orange (Bayferrox^®^ 960 and Bayferrox^®^ 4960) pigments.

Pigment	L*	a*	b*	*C*	*h*°
La_3_LiMnO_7_	69.5	+25.7	+63.8	68.8	68.1
La_3_LiMn_0.99_Ti_0.01_O_7_	68.7	+26.1	+64.4	69.5	67.9
La_3_LiMn_0.97_Ti_0.03_O_7_	67.2	+27.3	+65.4	70.9	67.3
Bayferrox^®^ 960	59.0	+21.0	+47.5	51.9	66.1
Bayferrox^®^ 4960	55.9	+23.5	+47.3	52.8	63.6

**Table 4:** L*a*b*Ch° color coordinate data of the La_3_LiMn_0.97_Ti_0.03_O_7_ pigment before and after the chemical stability test.

Treatment	L*	a*	b*	*C*	*h*°
As synthesized	67.2	+27.3	+65.4	70.9	67.3
4% CH_3_COOH	65.5	+27.0	+58.7	64.6	65.3
4% NH_4_HCO_3_	62.0	+26.9	+58.2	64.1	65.2

## Data Availability

Data is contained within the article.

## Sample Availability

Samples of the compounds are not available from the authors.
